# Supplementary figures and images for: Primary infection with dengue or Zika virus does not affect the severity of heterologous secondary infection in macaques
Source: PLoS Pathog. 2019 Aug 1;15(8):e1007766. doi: 10.1371/journal.ppat.1007766 (PMC6675051; doi:10.1371/journal.ppat.1007766)

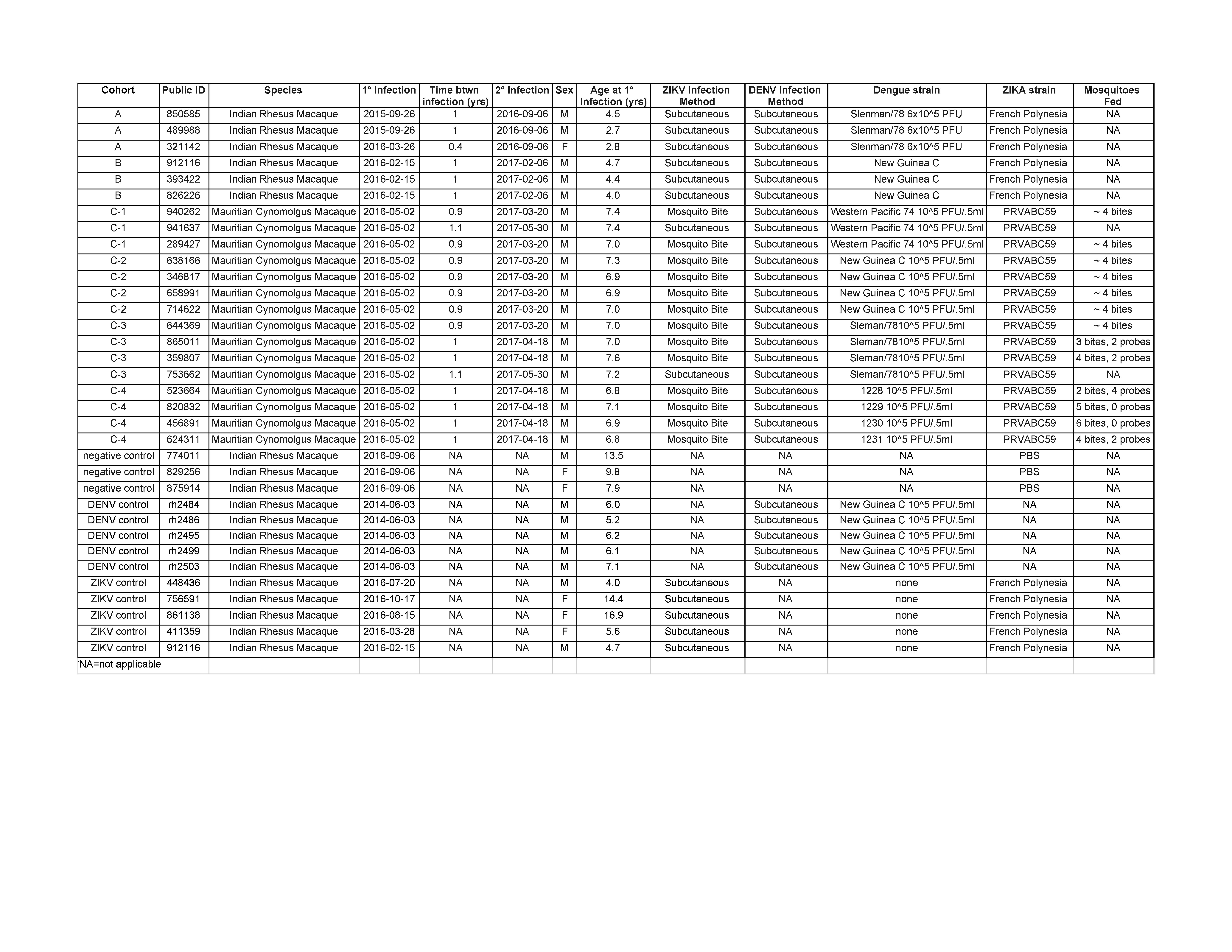

Supplement: S1 Table — (TIFF) [file ppat.1007766.s001.tiff]

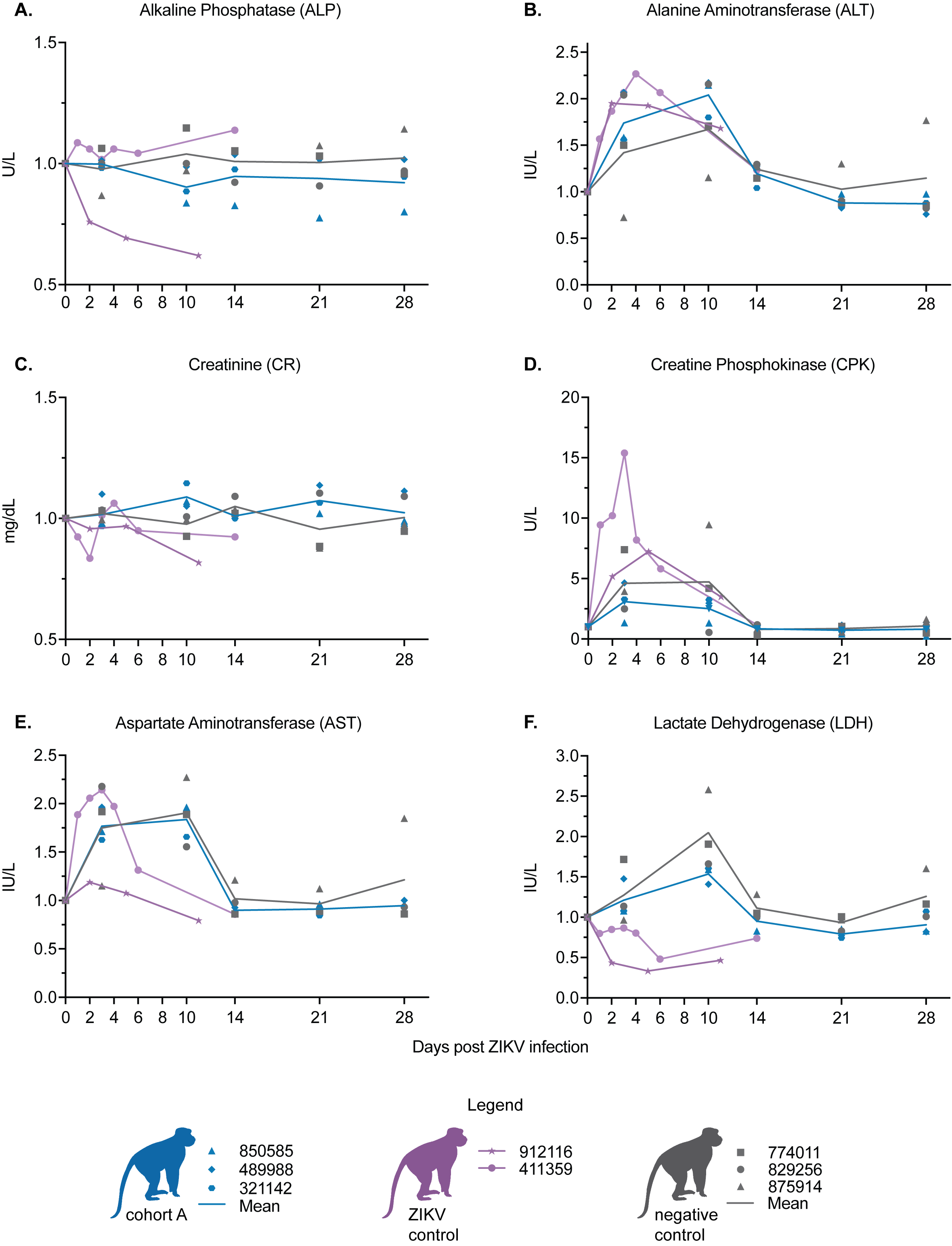

Supplement: S1 Fig — Serum chemistry parameters were normalized to baseline and the fold change from baseline is plotted for each animal longitudinally over the 28 day study with a line representing the mean of cohort A or the negative control group. The ZIKV control group contains two animals and lines are plotted for each animal rather than a mean. The Legend showing which symbol and color represents each animal is at the bottom of the figure. Parameters are plotted for (A) Alkaline Phosphatase (ALP) (B) Alanine Aminotransferase (ALT) (C) Creatinine (CR) (D) Creatine Phosphokinase (CPK) (E) Aspartate Aminotransferase (AST) and (F) Lactate Dehydrogenase (LDH). (TIF) [file ppat.1007766.s002.tif]

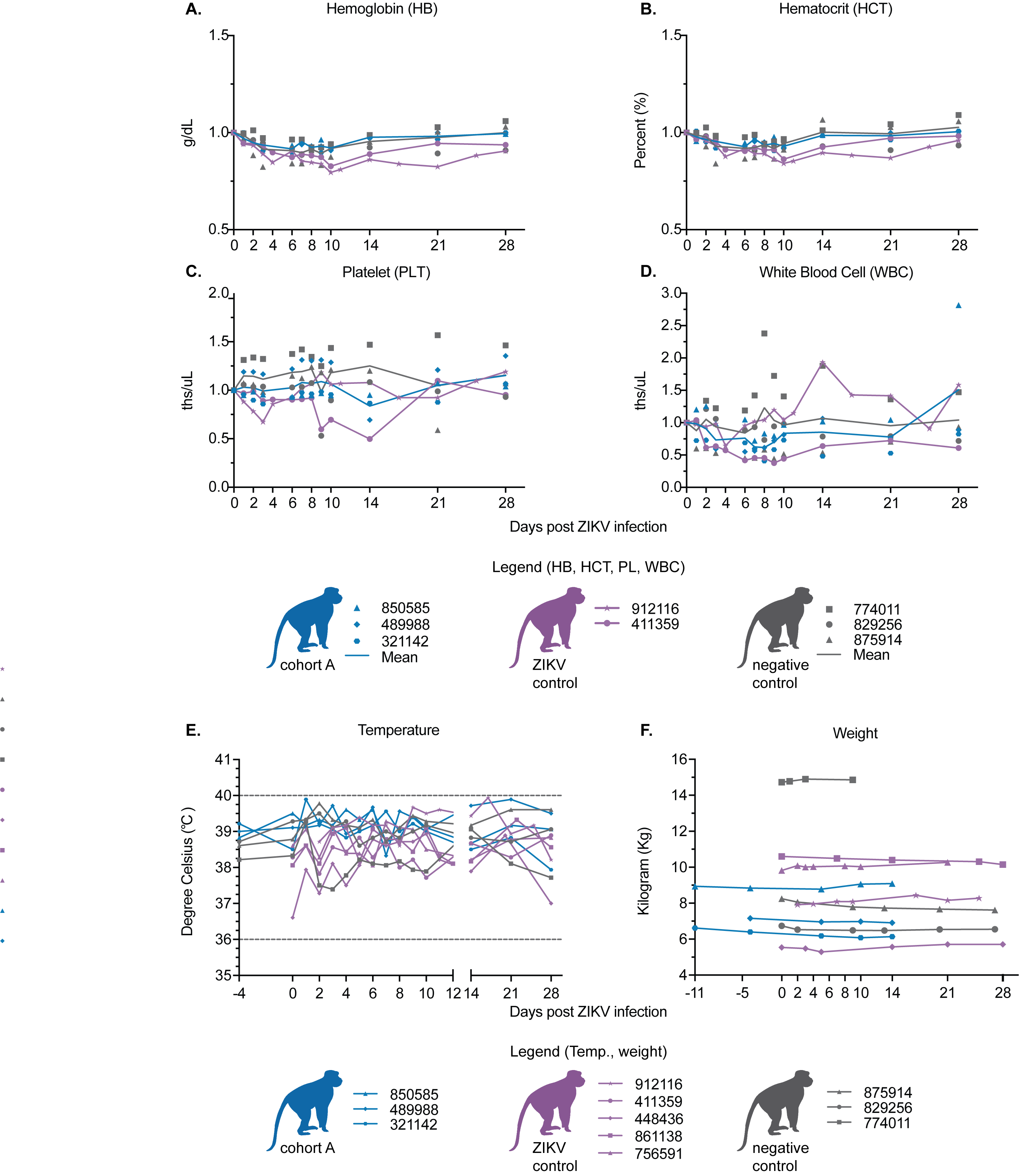

Supplement: S2 Fig — Complete Blood Counts (CBCs) were normalized to baseline and the fold change from baseline is plotted longitudinally for 28 dpi for (A) Hemoglobin (HB) counts (B) Hematocrit (HCT) (C) Platelet (PLT) counts and (D) White Blood Cell (WBCs) counts. Individual values are plotted for each animal with lines representing the mean for the cohort A and negative control animals. ZIKV control animals are each plotted independently with lines. The Legend for animals in panels A-D is located under panel C. (E) Temperatures of each animal plotted longitudinally over 28 days. Dotted lines represent the normal range of temperatures for Indian rhesus macaques at WNPRC. (F) Weight of each animal plotted longitudinally. The legend for panels E and F are located on the bottom of the figure. Three ZIKV control animals had weights and temperatures collected but not CBCs. (TIF) [file ppat.1007766.s003.tif]

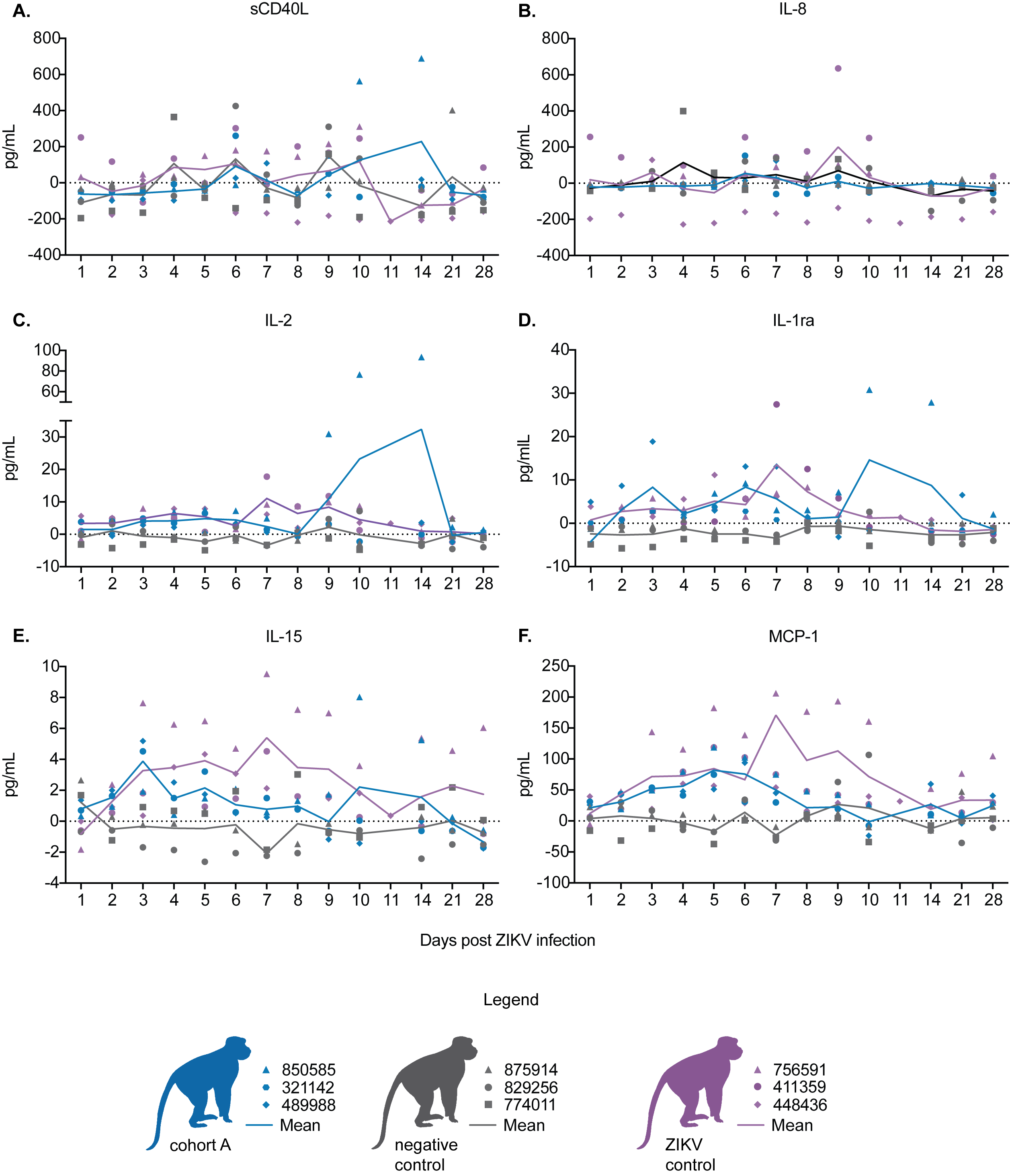

Supplement: S3 Fig — Cytokine concentrations detected by Luminex assay were normalized to baseline and the change from baseline was plotted longitudinally for (A) sCD40L (B) IL-8 (C) IL-2 (D) IL-1ra (E) IL-15 and (F) MCP-1. Lines represent the mean values for each group (see Legend). Individual values for each animal are also plotted. (TIF) [file ppat.1007766.s004.tif]

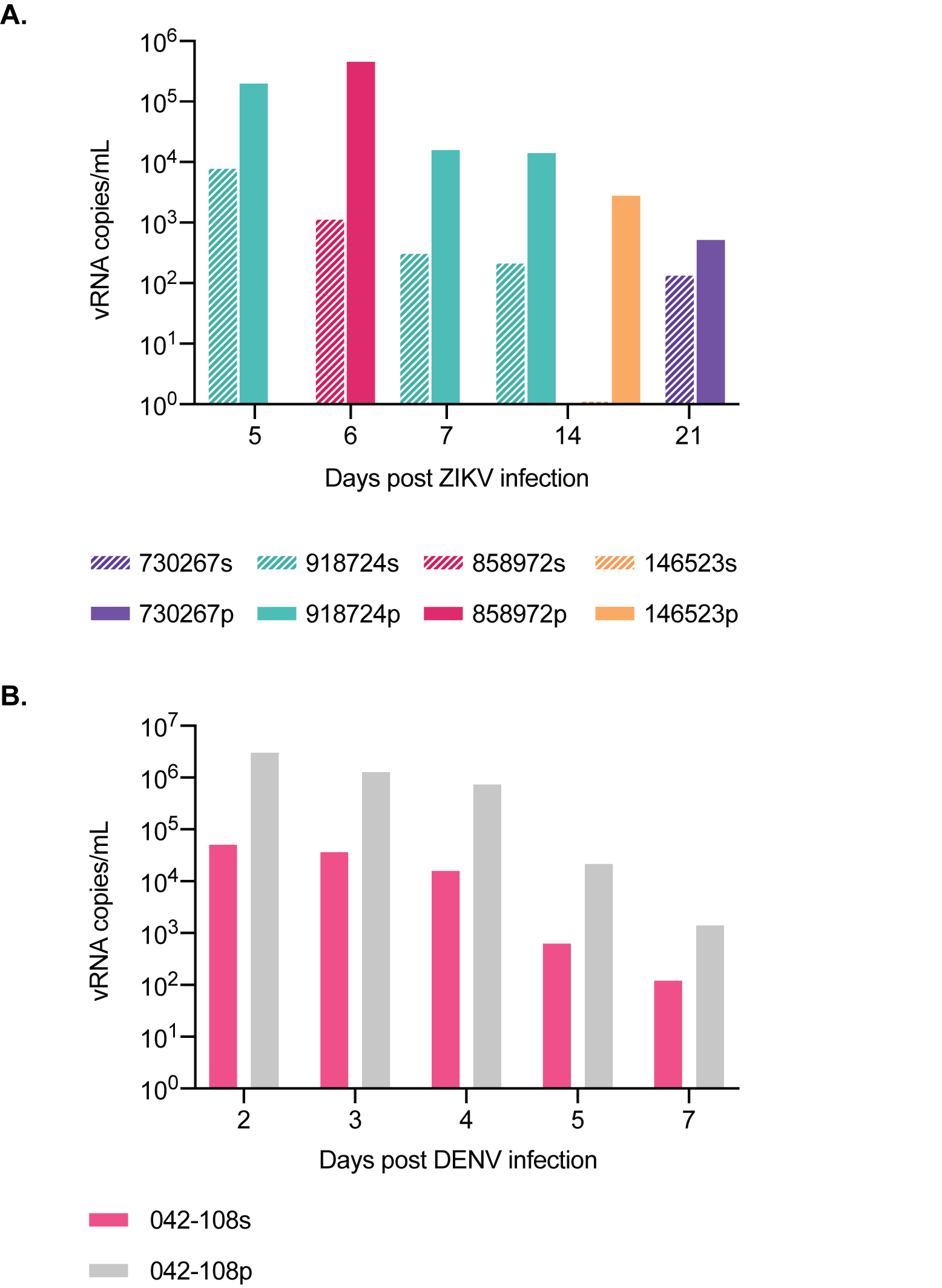

Supplement: S4 Fig — (A) ZIKV viral RNA copies/mL is shown for matched plasma (p) and serum (s) samples isolated from different animals at different time points after ZIKV infection. These animals were pregnant at the time of infection and therefore exhibit longer viremia than non-pregnant animals otherwise shown in this study (i.e. 21 dpi). (B) DENV viral RNA copies/mL isolated from matched plasma and serum samples from a single pregnant animal infected SC with 1x10^4^ PFU DENV-2/US/BID-V5945/2006. DENV viral loads were run as described for the ZIKV viral loads in the Methods section with a limit of detection of 100 vRNA copies/ml. (TIF) [file ppat.1007766.s005.tif]

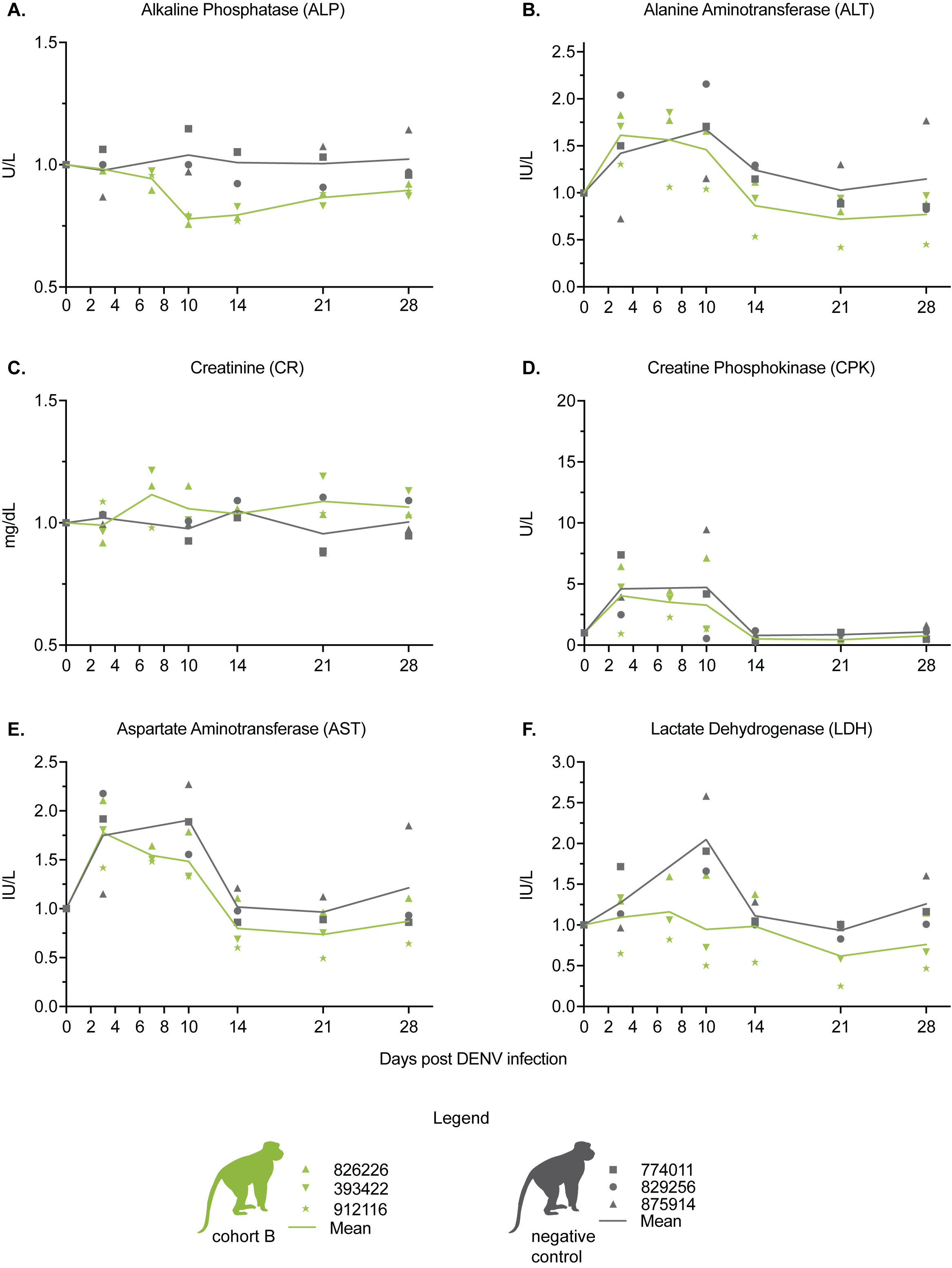

Supplement: S5 Fig — Serum chemistry parameters were normalized to baseline and the fold change from baseline is plotted for each animal longitudinally over the 28 day study with a line representing the mean of cohort B or the negative control group (see Legend). Parameters are plotted for (A) Alkaline Phosphatase (ALP) (B) Alanine Aminotransferase (ALT) (C) Creatinine (CR) (D) Creatine Phosphokinase (CPK) (E) Aspartate Aminotransferase (AST) and (F) Lactate Dehydrogenase (LDH). (TIF) [file ppat.1007766.s006.tif]

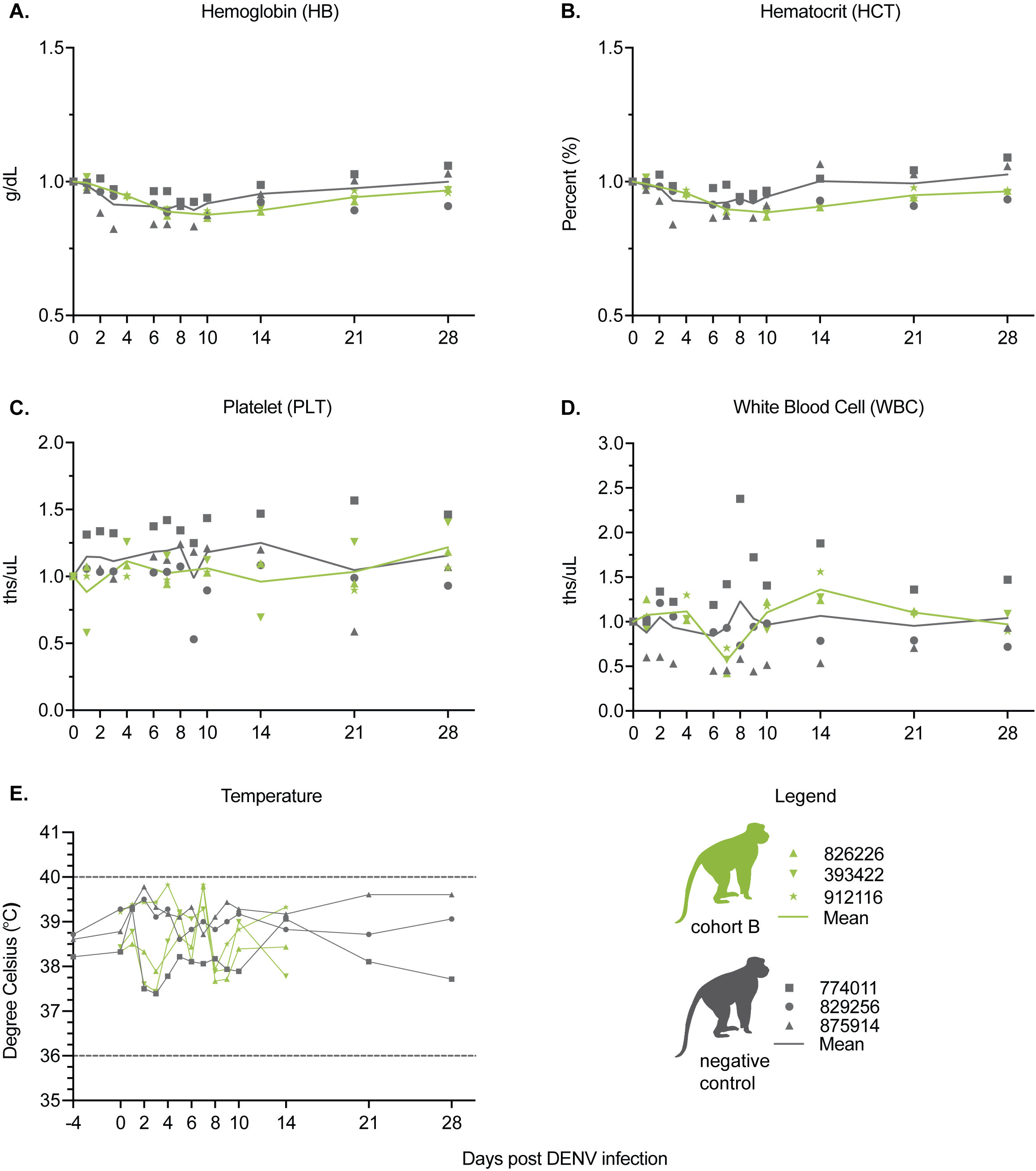

Supplement: S6 Fig — Complete Blood Counts (CBCs) were normalized to baseline and the fold change from baseline is plotted longitudinally for 28 dpi for (A) Hemoglobin (HB) counts (B) Hematocrit (HCT) (C) Platelet (PL) counts and (D) White Blood Cell (WBCs) counts. Individual values are plotted for each animal with lines representing the mean for the cohort B and negative control animals (see Legend). (E) Temperatures of each animal plotted longitudinally over 28 days. Dotted lines represent the normal range of temperatures for Indian rhesus macaques at WNPRC. (TIF) [file ppat.1007766.s007.tif]

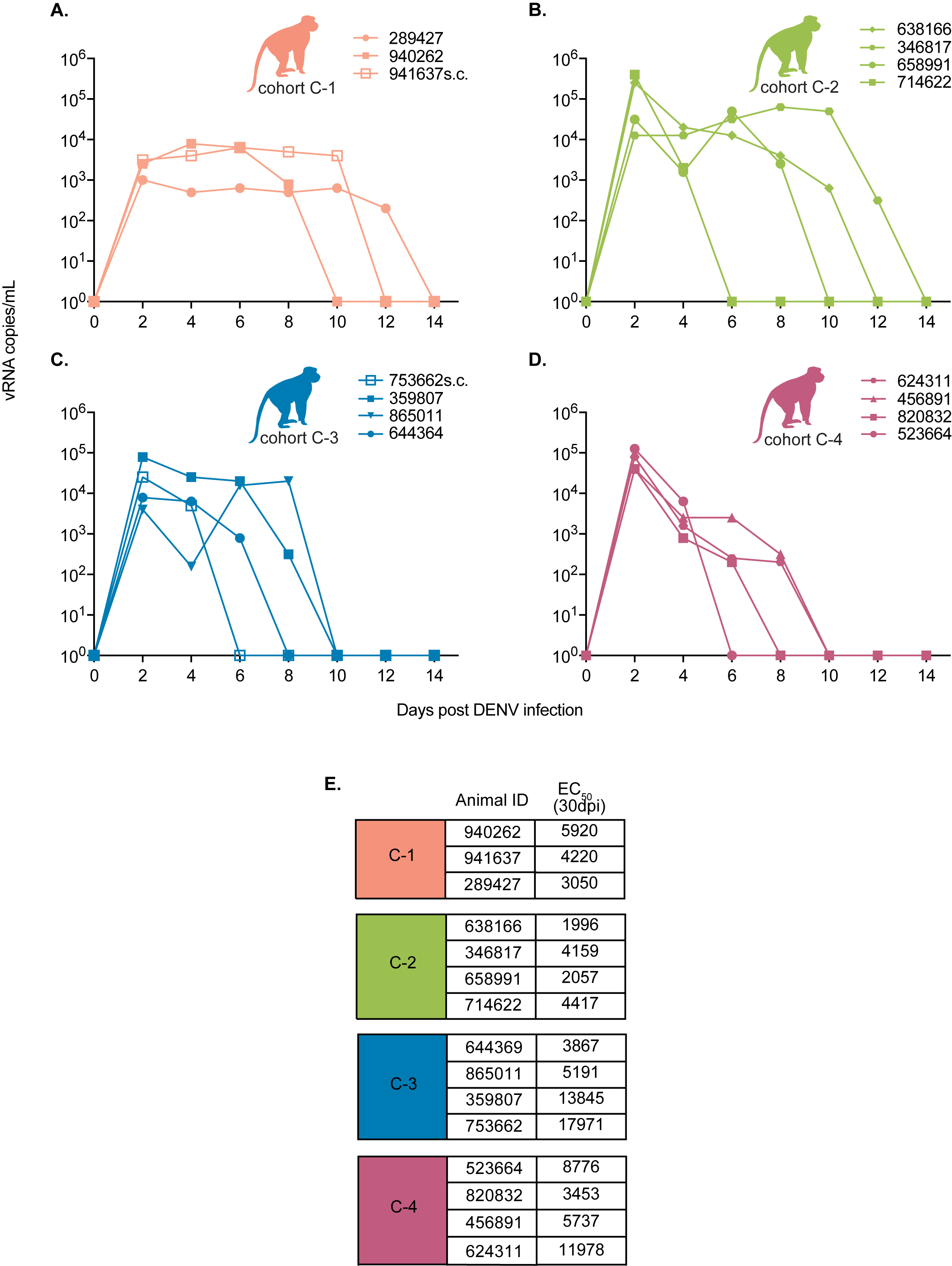

Supplement: S7 Fig — Longitudinal DENV RNA serum viral loads (copies/ml) in cohort C MCM after primary DENV infection. Cohort C 1–4 DENV antibody titer determined by a RVP neutralization assay performed at 30dpi. (A) Cohort C-1 animal with exposure to DENV-1 (B) Cohort C-2 animals with exposure to DENV-2. (C) Cohort C-3 animals with exposure to DENV-3. (D) Cohort C-4 animals with exposure to DENV-4. (E) DENV-specific RVP neutralization titers of cohort C animals at 30 days post-DENV infection. (TIF) [file ppat.1007766.s008.tif]

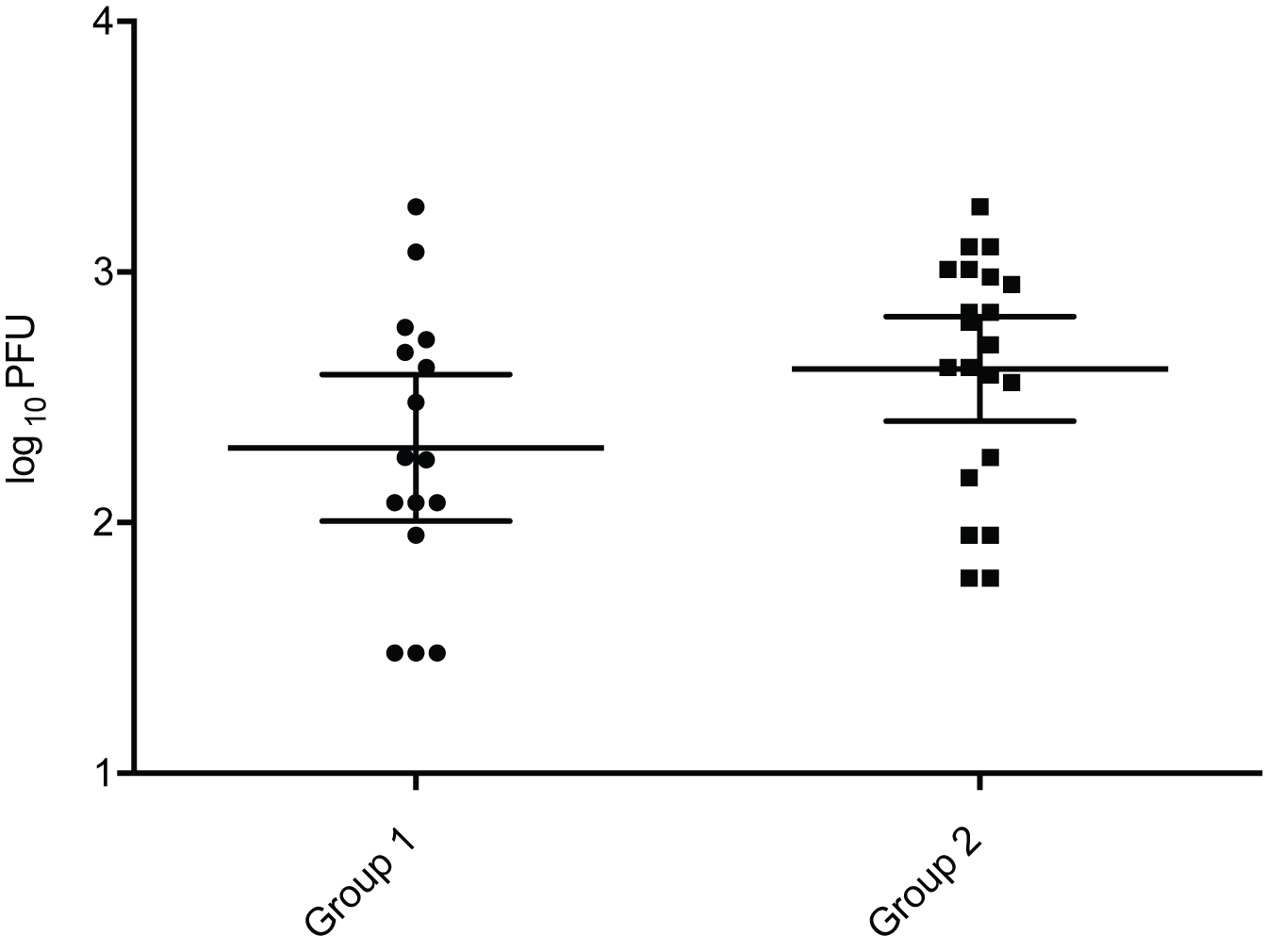

Supplement: S8 Fig — MCM were divided into two groups for mosquito inoculation. Animals in Group 1 include 940262, 289427, 638166, 346817, 658991, 714622, and 644369. Animals in Group 2 include 941637, 865011, 359807, 753662, 523664, 820832, 456891, and 624311. Mosquitoes were infected by feeding on ZIKV-infected mice and saliva from mosquitos used to infect each group who took a blood meal was expectorated from the mosquito after the MCM feeding session. Plaque forming units were quantified from the saliva to estimate the dose of ZIKV delivered by each mosquito bite. 941637 and 753662 were not infected after mosquito bite challenge and were subsequently infected subcutaneously. (TIF) [file ppat.1007766.s009.tif]

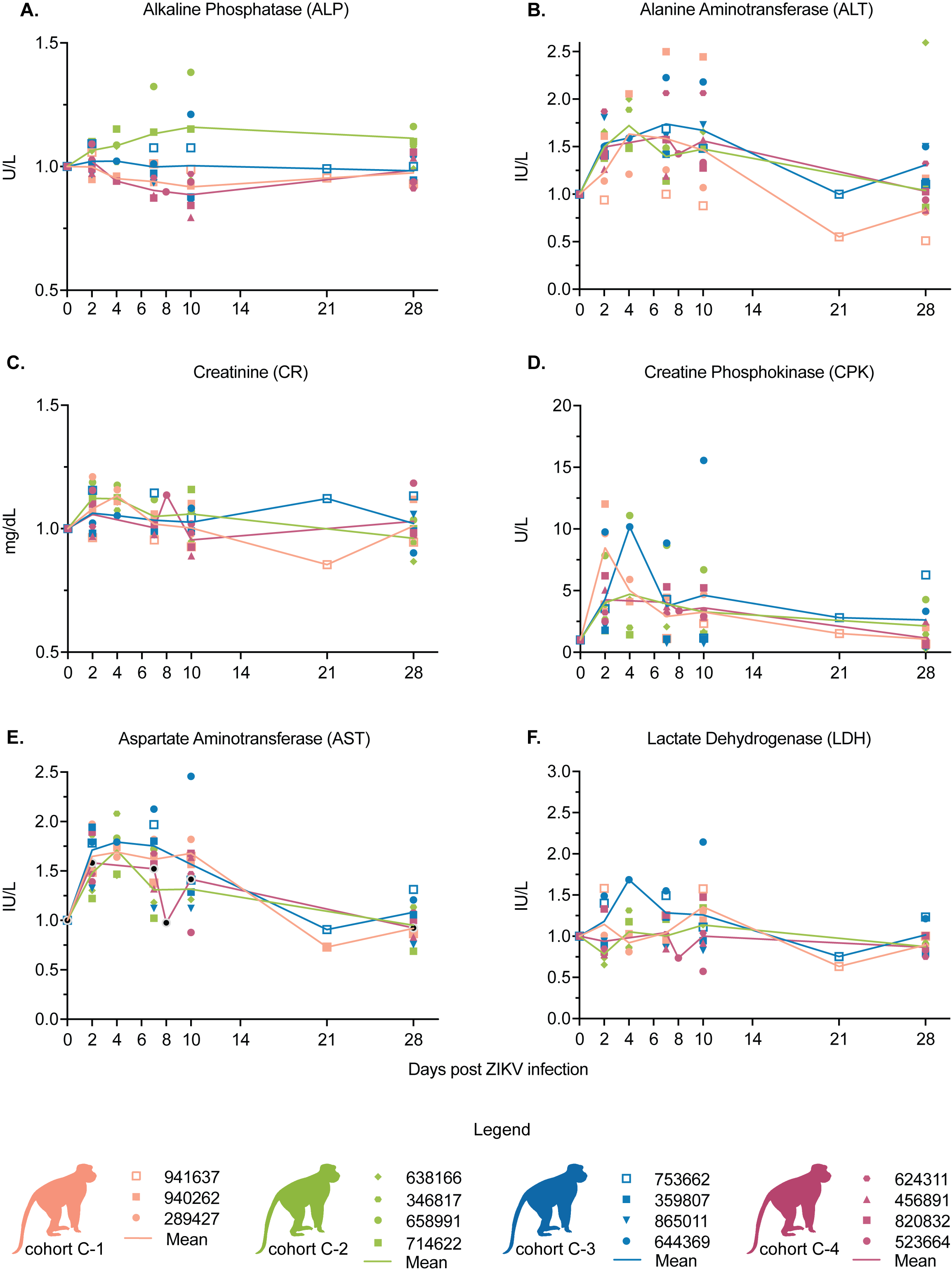

Supplement: S9 Fig — Serum chemistry parameters were normalized to baseline and the fold change from baseline is plotted for each animal longitudinally over the 28 day study with a line representing the mean for each group of cohort C animals (see Legend). Cohort C-1 animals were previously exposed to DENV-1, C-2 to DENV-2, C3 to DENV-3 and C4 to DENV4. Parameters are plotted for (A) Alkaline Phosphatase (ALP) (B) Alanine Aminotransferase (ALT) (C) Creatinine (CR) (D) Creatine Phosphokinase (CPK) (E) Aspartate Aminotransferase (AST) and (F) Lactate Dehydrogenase (LDH). (TIF) [file ppat.1007766.s010.tif]

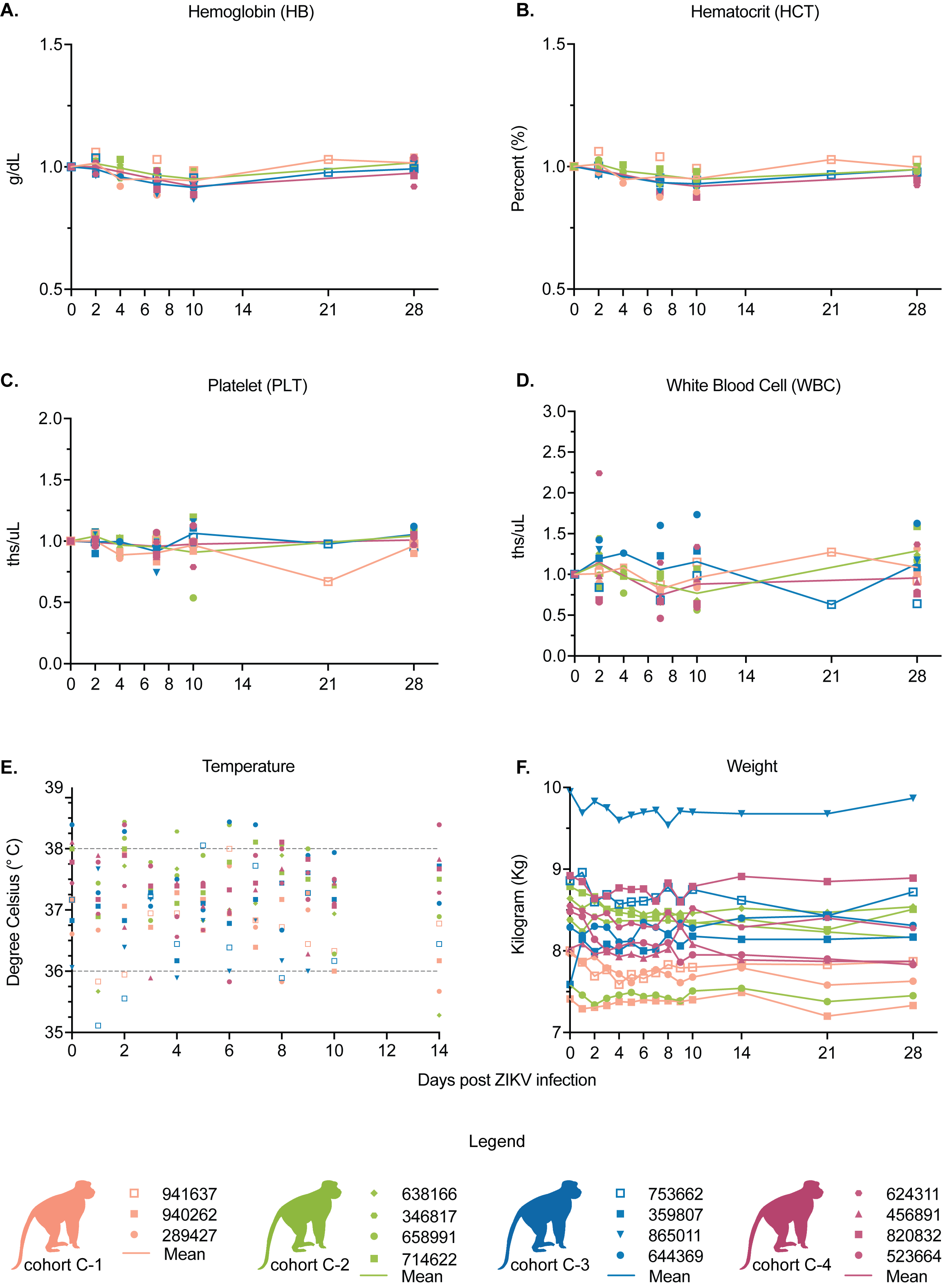

Supplement: S10 Fig — Complete Blood Counts (CBCs) were normalized to baseline and the fold change from baseline is plotted longitudinally for 28 days after infection for (A) Hemoglobin (HB) counts (B) Hematocrit (HCT) (C) Platelet (PLT) counts and (D) White Blood Cell (WBCs) counts. Individual values are plotted for each animal with lines representing the mean for each cohort C group (see Legend). (E) Temperatures of each animal plotted longitudinally over 28 days. Dotted lines represent the normal range of temperatures for cynomolgus macaques at WNPRC. (F) Weight of each animal plotted longitudinally. (TIF) [file ppat.1007766.s011.tif]
